# Receptor-Mediated Vascular Smooth Muscle Migration Induced by LPA Involves p38 Mitogen-Activated Protein Kinase Pathway Activation

**DOI:** 10.3390/ijms10073194

**Published:** 2009-07-13

**Authors:** Zhi-Bin Zhou, Jian-Ping Niu, Zhi-Jun Zhang

**Affiliations:** 1 School of Clinical Medicine, Southeast University, Nanjing 21009, China; E-Mail: zhouzhibin74@163.com (Z.-B.Z.); 2 Department of Neurology, The second Hospital of Xiamen, Xiamen 361021, China

**Keywords:** lysophosphatidic acid, receptor, vascular smooth muscle cell, migration, p38 mitogen-activated protein kinase

## Abstract

Lysophosphatidic acid (LPA), a naturally occurring glycerophospholipid, can evoke various biological responses, including cell migration, proliferation and survival, via activation of G protein-coupled receptors (GPCRs). However, the role of LPA receptors and details of LPA signaling in migration are largely unexplored. In this study we detect the expression of LPA1 and LPA3 receptors in rat aortic smooth muscle cells (RASMCs). LPA stimulated RASMCs migration in a dose-dependent manner and induced the phosphorylation of p38 mitogen-activated protein kinase (p38MAPK) and extracellular signal-regulated kinase (ERK). LPA-induced cell migration was significantly inhibited by specific LPA1/LPA3-receptor antagonist Dioctylglycerol pyrophosphate (8:0) (DGPP8.0) at higher concentration. Migration of cells toward LPA was partially, but significantly, reduced in the presence of SB-203580, a p38 MAPK inhibitor, but not PD98059, an ERK inhibitor. In addition, pertussis toxin (PTX), a Gi protein inhibitor, induced an inhibitory effect on p38 MAPK, ERK phosphorylation and RASMCs migration. These data suggest that LPA-induced migration is mediated through the Gi-protein-coupled LPA1 receptor involving activation of a PTX-sensitive Gi / p38MAPK pathway.

## 1. Introduction

Lysophosphatidic acid (LPA) is a member of the lysophospholipid class of phospholipids that is an intermediate in phospholipid metabolism [1]. In addition to being a key intermediate in *de novo* lipid synthesis, LPA is also an important intercellular messenger, which can act as either an autocrine or paracrine mediator. Originally reported to be the primary phospholipid growth factor in mammalian serum [2,3], it is now known to be a mediator of diverse cellular processes, such as migration [4–6], proliferation and cell survival [7,8], aggregation of platelets [9,10], smooth-muscle contraction [11,12], cytoskeletal reorganization [13–14], myelination [15,16], neurogenesis [17,18] and neurotransmitter release [19,20].

LPA elicits these cellular effects on most cell types through the activation of its specific G protein–coupled receptors (GPCRs). At least six LPA-specific mammalian GPCRs, LPA1-6, have been identified to date. Among the six LPA receptors, LPA1 [21], LPA2 [22] and LPA3 [23] are members of the endothelial differentiation gene (EDG) family, share about 50% amino acid sequence identities. The other three LPA receptors LPA4/p2y9 [24], LPA5/GPR92 [25,26], LPA6/GPR87 [27], which show small similarities with the Edg family GPCRs, were recently identified and comparatively less is known about these receptors. LPA1 is the receptor with the widest distribution, the expression of LPA2 and LPA3 is somewhat more restricted, whereas LPA4 is expressed only in the ovary [24], LPA5 is mainly expressed in the lymphocyte compartment of the gastrointestinal tract, sensory dorsal root ganglia as well as embryonic stem cells [25,26], LPA6 was expressed in placenta, ovary, testis, prostate, brain, and skeletal muscle [27].

When an agonist interacts with a specific GPCR, its associated G-protein is activated and induces a specific intracellular pathway that leads to the final cellular response. At least three different heterotrimeric G-proteins have been associated with the actions of LPA in various cell types: Gi/o, Gq/11, and G12/13 [28,29].

The migration of vascular smooth muscle cells (VSMCs) is believed to play a major role in the pathogenesis of atherosclerosis and is the main cause of restenosis after balloon angioplasty. Elucidation of the mediators and knowledge of their mode of action may provide useful information for the development of therapeutic treatments for these diseases [30]. In VSMCs, LPA has been shown to stimulate migration [31] and proliferation [32]. Results derived from LPA receptor knockout mice illustrate that LPA1^−/−^2^−/−^ SMCs exhibit decreased migration in response to LPA, whereas LPA1^−/−^ SMCs exhibit enhanced migration in response to upregulation of the LPA3 receptor [33]. Damirin A *et al* [34] demonstrated that LPA1 receptors are involved in the LDL-induced migration of human coronary artery smooth muscle cells. However, the roles of LPA receptors in LPA-stimulated VSMCs migration are far from been elucidated in detail. MAPKs are believed to be associated with the migration and proliferation of VSMCs [35,36], but which subfamily of MAPKs is involved in VSMCs remains controversial. The present study was designed to determine the involvement of LPA receptors in LPA-stimulated migration of VSMC and the signaling pathways involved.

## 2. Results and Discussion

### 2.1. RASMCs Migration toward LPA

**Figure 1.**
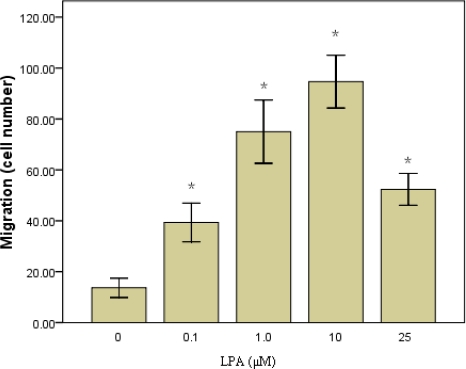
LPA-induced migration of RASMCs. RASMCs were stimulated with the indicated concentrations of LPA and migration was determined using the Boyden chamber assay. Values are means ±S.E.M n =3. *P<0.01 vs. control (0μM LPA).

In order to confirm that LPA was able to induced RASMCs migration in our model, we performed a cellular migration assay. RASMCs (1x10^5^ cells) were added to the upper wells of the Boyden chamber containing LPA (0–25 μM) in the lower chamber. Cells were incubated for 6 h to allow to migration. Results show that cells were induced by LPA to migrate to the lower well in a dose-dependent manner. The greatest number of migrating cells occurred at 10 μM LPA. The number of migrating cells decreased at higher LPA concentrations (Figure 1).

### 2.2. LPA Receptor Expression

**Figure 2.**
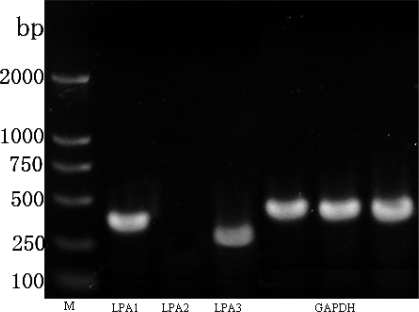
LPA receptor expression in RASMCs. Total RNA was isolated and RT-PCR was performed to determine LPA receptor expression.

As we have demonstrated that LPA can induce RASMCs migration, it was then essential to determine which LPA receptor may be responsible. Up to date, six receptors have been described, whereas LPA4-6 are not expressed in vascular tissue. Therefore we performed Semi-quantitative RT-PCR analysis to determine mRNA expression levels of the LPA receptors(LPA1-3) in RASMCs. Consistant with previous results [37], LPA1 and LPA3 were expressed in RASMCs, whereas LPA2 expression could not be detected (Figure 2).

### 2.3. LPA-Induced Migration in RASMCs Is Mediated by LPA1 Receptor

**Figure 3.**
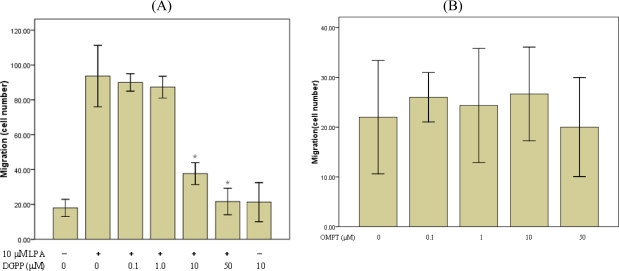
Involvement of specific LPA receptor subtypes in LPA-induced migration. RASMCs were pretreated with the indicated concentrations of (A) DGPP (0–50 μM) (B) OMPT (0–50 μM) for 1 h. Cellular migration assays were then performed in the presence or absence of or LPA (10μM). Values are means ±S.E.M n =3. *P<0.01 vs. control (LPA alone).

To characterize the receptors involved in LPA-induced migration of RASMCs, cells were treated with varying concentrations of the LPA1 and LPA3 antagonist, DGPP8:0, and LPA3 agonist, OMPT in the presence or absence of LPA (10 μM). The migration response to LPA was significantly inhibited by DGPP8:0 at 10 μM and was almost completely inhibited at 50μM. However, low concentrations of DGPP (0.1–1μM) and all concentrations of OMPT (up to 50μ M) had little effect on LPA-induced migration (Figure 3). DGPP has been shown to block LPA3 and LPA1 receptors with a Ki of 106 nM and 6.6 μM, respectively [38]. Therefore, these data suggest that LPA induced RASMCs migration may be mediated through the LPA1 receptor.

### 2.4. P38MAPk Pathway Activation Is Required in LPA-Induced Migration

**Figure 4.**
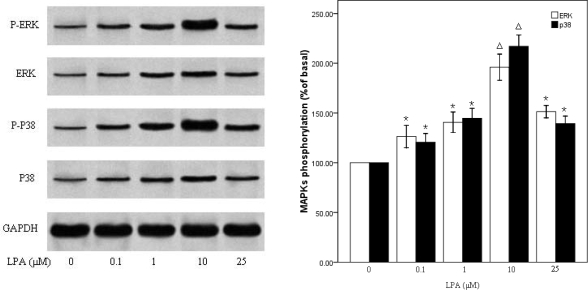
LPA induced phosphorylation of p38 MAPK and ERK. (Left panel) RASMCs were stimulated with the indicated concentrations of LPA for 10min. Total cell lysate was resolved by SDS-PAGE and immunoblotted with the indicated antibodies. (Right panel) Densitometric quantification of MAPK phoshorylation. P38MAPK or ERK activation was determined as an increase in the ratio of phospho-MAPK/total MAPK. Values are means ±S.E.M n =3. *P<0.01 vs. control (0μM LPA), ΔP<0.01 vs. each other group.

**Figure 5.**
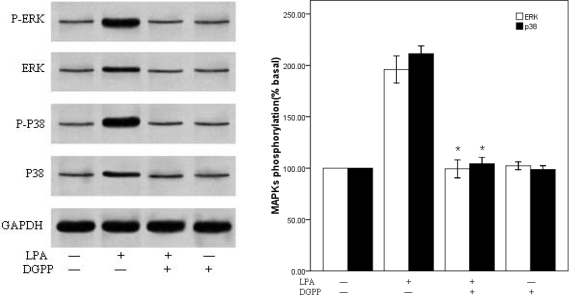
Effect of DGPP on p38 MAPK and ERK phosphorylation. A: (Left panel) RASMCs were pretreated with vehicle or DGPP (50μM) for 30 min, then stimulated with 10μM LPA or vehicle for 10 min. Total cell lysate was resolved by SDS-PAGE and immunoblotted with the indicated antibodies. (Right panel): Densitometric quantification of MAPK phoshorylation. P38MAPK or ERK activation was determined as an increase in the ratio of phospho-MAPK/total MAPK. Values are means ±S.E.M n =3. *P<0.01 vs. control (LPA alone).

**Figure 6.**
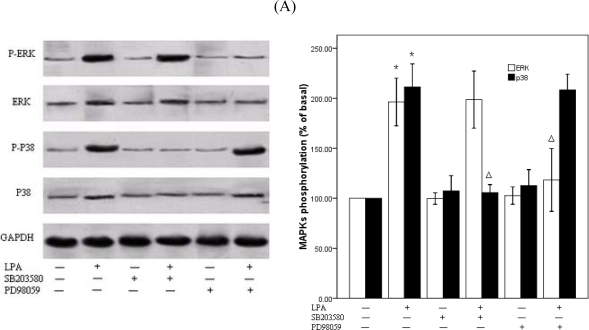

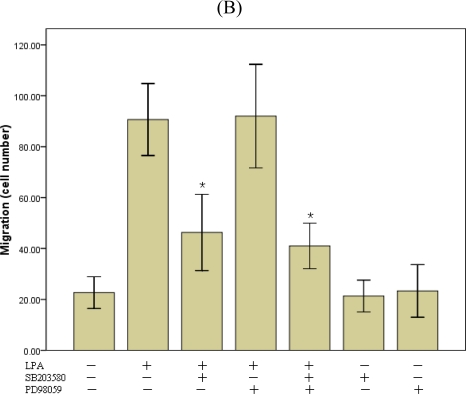
Effects of p38 and ERK inhibition on LPA-induced migration of RASMCs. A: (Left panel) RASMCs were pretreated with vehicle, SB203580 (30μM) or PD98059 (30μM) for 30 min, then stimulated with 10 μM LPA or vehicle for 10 min. Total cell lysate was resolved by SDS-PAGE and immunoblotted with the indicated antibodies. (Right panel) Densitometric quantification of MAPK phoshorylation. P38MAPK or ERK activation was determined as an increase in the ratio of phospho-MAPK/total MAPK. *P<0.01 vs. control (vehicle), Δ P<0.01 vs.control (LPA alone). B: RASMCs were pretreated with SB203580or/and PD98059 then treated with 10 μM LPA. Cellular migration assays were performed. Values are means ±S.E.M n =3. *P< 0.05 vs. control (LPA alone).

We next wanted to further elucidate the molecular cascades involved in LPA-induced migration of RASMCs. Cells were treated with (0– 25 μM) LPA for 10 min and Western blot analysis performed to determine the activation of specific kinase signaling pathways using specific antibodies to detect the phosphorylated forms of p38MAPKand ERK. LPA induced an increase in the phosphorylation of p38 MAPK and ERK in a dose-dependent manner, which reached peak at 10 μM LPA, approximately 2-fold above the basal level, and then decreased (Figure 4). We next examined the effects of DGPP on the activity of ERKand p38MAPK. RASMCs were pretreated with DGPP (50 μM) for 30 min and then stimulated with 10 μM LPA for 10 min. The phosphorylation of ERK and p38MAPK were completely blocked by pretreatment with DGPP (Figure 5). To investigate the possible involvement of p38MAPK or ERK phosphorylation in LPA-induced RASMCs migration, we examined the effects of specific inhibitors for p38 MAPK and ERK kinases on cell migration. RASMCs were pretreated with a p38MAPK inhibitor, SB203580 (30 μM), and an ERK inhibitor, PD98059 (30 μM), for 30 min, and then stimulated with 10 μM LPA for 10 min. Cellular migration in response to LPA was analyzed. LPA-induced p38MAPK activation was attenuated to the base level by pretreatment with SB203580 (Figure 6A). Migration assays show that a partial, but significant, reduction of migrated cells from 91±6 (n =3) in the absence of SB203580 to 46 ±6 (n= 3) in the presence of SB203580 (Figure 6B). Pretreatment of the cells with PD98059 almost completely abolished the LPA-induced phosphorylation of ERK, but, in contrast to SB203580, it failed to block LPA-induced VSMC migration (Figures 6A and B). When SB203580 and PD98059 were coadministered, the inhibiting action of SB203580 on the RASMCs migration were not strengthened (Figure 6B).

### 2.5. Involvement of Gi Protein in LPA-Mediated Migration

**Figure 7.**
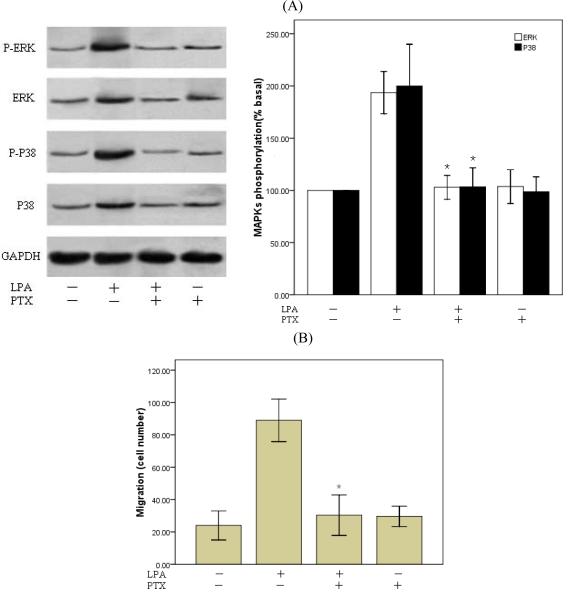
LPA induces RASMCs migration through Gi pathway. RASMCs were pretreated overnight with 100 ng/mL PTX and then stimulated with 10 μM LPA or vehicle for 10 min. A: (Left panel) Total cell lysate was resolved by SDS-PAGE and immoblotted with the indicated antibodies. (Right panel) Densitometric quantification of ERK and p38 phosphorylation. *P<0.01 vs. control (LPA alone). B: RASMCs migration assay.*P<0.01 vs. control (LPA alone). Values are means ±S.E.M n =3.

Three G-proteins have been previously shown to play a role in LPA signaling, Gi/o, Gq/11, and G12/13 [28,29]. LPA stimulates cell proliferation through activation of tyrosine kinase and MAP kinase, Gi/o-type proteins are the most likely candidates to mediate these effects [39]. And results derived from LPA receptor knockout mice illustrate that at least in mouse embryonic fibroblasts (MEFs), LPA1 receptors is primarily coupled to Gi [28]. So we employed PTX, a specific inhibitor of Gi/o to assess the role of G proteins in LPA-induced RASMCs migration. Overnight treatment (12h–14h) of the cells with PTX (100 ng/ml) prevented LPA-induced phosphorylation of p38 MAPK and ERK(Figure 7A) and almost completely abolished LPA-induced migration (Figure 7B). These data suggest that the Gi-activated pathways are upstream of p38 and ERK activation and required for LPA-induced RASMCs migration.

LPA is released from activated platelets, injured cells, and growth factor-stimulated cells and has been shown to accumulate in the neointima of human atherosclerotic plaques [40]. It can also stimulate the proliferation [31] and migration [32] of VSMCs, thus it is considerably relevant to vascular disease, such as atherosclerosis [40,41] and vascular remodeling *in vivo* [32]. Although the effects of LPA have been widely studied, little is known about the role of LPA receptors and its signaling pathways activated by LPA in LPA-stimulated VSMCs migration. The present study shows that specific LPA receptor subtypes mediate LPA-induced migration in RASMCs through p38 MAPK activation.

A large body of evidence suggests that LPA induces cell migration in various cell lines [4–6,41]. In agreement with these results, we observed that LPA induces the migration of RASMCs in a dose-dependent manner. Furthermore, we found that the greatest number of migrating cells occurred at 10 μM LPA and then dropped at higher dose. The reason for this may be that RASMCs were not sensitive or tolerant to the LPA stimulation at higher concentration of LPA. It is generally accepted that the diverse effects of LPA are mediated by its receptors. Receptor-mediated actions of LPA have important influences on cell survival, cytoskeletal remodeling, cell migration, and cell proliferation. In the present study, we demonstrate that RASMCs expressed two types of LPA receptor transcripts (LPA1 and LPA3) (Figure 2), and LPA-induced migration in RASMCs is mediated by specific LPA receptors. Although both LPA1 and LPA3 are expressed, data suggests that LPA1, not LPA3, is required for LPA-induced RASMCs migration. Pretreatment of cells with an LPA1/LPA3-receptor antogonist, DGPP, prevented LPA-induced RASMCs migration. DGPP blocks LPA1 with a Ki of 6.6 μM versus LPA3 with a Ki of 106 nM [36]. Lower concentrations of DGPP (0.1–1 μM), which inhibit only LPA3, were relatively ineffective at blocking cell migration. However, at concentrations more than 10 μM, which inhibit both LPA1 and LPA3, there was a significant reduction in RASMCs migration. This was further confirmed by data demonstrating that the LPA3 selective agonist, OMPT, hardly affected LPA-induced migration. However, since no specific LPA1 antagonist or agonist being available, this conclusion remains to be validated by further study with specific intervention on LPA1 under our experimental conditions.

LPA receptors couple to at least three G proteins including Gq, Gi, and G12/13, LPA1 and LPA2 can couple to the Gi /o, G12/13, and Gq family, and LPA3 can couple to the Gi /o, and Gq family [42]. LPA induces various biological effects through these PTX-sensitive (Gi /o) or -insensitive (G12/13, Gq) G-proteins. The Hirshman and Emalan groups reported [43] that antisense oligonucleotide depletion of both Gqα and Gαi-2 proteins significantly inhibited LPA-induced actin reorganization in airway smooth muscle. These data indicate that LPA-induced actin reorganization, which is a fundamental process in cell motility and division, is mediated by both Gi and Gq pathways. In the present study, the migration response to LPA was completely inhibited by PTX treatment. This finding suggests that LPA acting via a receptor that couples to the Gi-protein to induce RASMCs migration. In contrast, Kim *et al.* [44] indicated that LPA coupling to Gq is the primary pathway that determines VSMCs migration and proliferation, however, they were also able to see some component of LPAGi-mediated signaling in the mouse VSMCs. This may be possibly due to the conditions for cell migration *in vitro* are very different, cells always interact with the matrix. The extracellular matrix might explain the discrepancy, while their assay used a Boyden chamber apparatus like our chemotaxis assays, the filters on which the cells migrated were coated with fibronectin. The underlying mechanism of the discrepant observations remains to be elucidated.

Data from a variety of laboratories have indicated that several signal transduction molecules, including Src, phosphoinositide-3 kinase, and focal adhesion kinase, participate in the regulation of migration [45–47]. Mitogen-activated protein kinases (MAPKs), including p38 MAPK and ERK, have been shown to be activated downstream of GPCR. In addition, MAPKs have also been shown to be involved in regulating the migration and proliferation of VSMCs [35,36]. But there are conflicting views on which subfamily of MAPKs is involved in the intracellular signal pathway for cell migration. To check which signaling mechanism is involved in the regulation of LPA-induced RASMCs migration, we treated cells with different inhibitors. In the present study, LPA stimulated phosphorylation of p38 MAPK and ERK in RASMCs in a dose-dependent manner, which was in keeping with the migration assay. DGPP treatment completely abolished the LPA-induced p38MAPK and ERK activation, indicating that the LPA mediated the ERK and p38MAPK signaling pathways through LPA receptors. SB203580 treatment completely abolished the LPA-induced p38MAPK activation and, partially, but significantly, decreased migratory response to LPA. In contrast, although PD98059 treatment completely abolished LPA-induced ERK activation it did not influence LPA-induced migration. These data suggest a critical role for p38 MAPK, not ERK, in LPA induced RASMCs migration.

A growing body of evidence suggests that p38 MAPK is involved in cell migration by remodeling the actin cytoskeleton [48,49]. P38MAPK acts as an upstream of the protein kinases that lead to the phosphorylation of Hsp27 in various types of cells. The activation of Hsp27 plays a key role inmodulating actin polymerization and the cytoskeletal remodeling associated with VSMCs migration in response to stimuli such as PDGF, IL-1β, TGF-β and sphingosine 1-phosphate [50,51]. In the present study, LPA stimulated phosphorylation of p38 and ERK in RASMCs, but the migration was only affect by p38MAPK inhibiter. Although in some studies, ERK activation has been described as a necessary event in VSMC migration, there are also studies citing no effect of ERK inhibition so as to our study, or even ERK-mediated inhibition of migration [52]. This may suggests that ERK kinase can, and probably does, participate in VSMC migration but that there is redundancy in the signaling networks that compensates for ERK inhibition. LPA-induced increase of p38 MAPK phosphorylation is also significantly inhibited by treatment with PTX, which demonstrates that activation of p38 MAPK by LPA is mediated through Gi-protein activation. Further experiments are required to elucidate the interactions between Gi protein and p38 MAPK.

The involvement of a small G protein Rho in migratory activity has also been extensively examined in the previous studies of vascular SMCs, but its role remains controversial [31,35,53,54]. Ai *et al.* reported that C3 toxin, a specific inhibitor of Rho, and Y-27632, a Rho kinase or ROCK inhibitor, have blocked vascular SMC migration induced by PDGF, and LPA, suggesting the positive involvement of Rho or Rho kinase in SMC migration [31]. However, some results of experiments also reported that Rho plays an inhibitory role in SMC migration [53,54]. The inhibitory role of Rho in cell migration has also been reported in other cell systems [55,56]. Further experiments are required to understand the role of intracellular signaling pathways, including the Rho signaling pathway, involved in the LPA receptor-mediated regulation of migration of SMCs.

## 3. Conclusions

We have demonstrated that LPA can stimulate RASMCs migration through the Gi-protein-coupled LPA1 receptor. LPA1-Gi-p38 signal cascade is important for VSMC migration in response to LPA. Inhibition of these pathways might be advantageous for controlling VSMCs migration associated with vascular diseases such as arteriosclerosis and restenosis.

## 4. Experimental Section

### 4.1. Materials

1-Oleoyl-*sn*-glycero-3-phosphate (18:1LPA) and 1-oleoyl-2-*O*-methyl-*rac*-glycero-phosphothionate (OMPT) were obtained from Sigma–Aldrich. Dioctylglycerol pyrophosphate (8:0) (DGPP 8:0) was from Avanti Polar Lipids. Pertussis toxin (PTX) was from Alexis Biochemicals Corporation. Anti- p38 mitogen-activated protein kinase (p38 MAPK), anti-phosphorylated p38 MAPK, anti-extracellular signal-regulated kinase (ERK), anti-phosphorylated ERK, SB203580 and PD98059 were from Santa Cruz Biotechnologies.

### 4.2. Cell Culture

VSMCs were prepared from thoracic aorta of Sprague-Dawley rats, as previously described [57]. Subcultured cells from passages 3–5 were used and demonstrated 95% positive immunostaining with smooth muscle α-actin antibody. For the experiments, VSMCs at 80–90% confluency were used after serum depletion with serum-free Dulbecco’s modified Eagle’s medium for 2–3 days.

### 4.3. Determination of LPA Receptor Expression

Total RNA was isolated from cultured VSMCs with Trizol according to the manufacturer’s protocol. RT-PCR was performed to determine the mRNA expression level of the LPA receptor subtypes (LPA1, LPA2, LPA3). Expression levels were normalized by GAPDH mRNA levels. The specific primer sets for rat LPA1, LPA2 and LPA3 were as follows: LPA1 Fwd 5′-GGAAGTATGTTTGTGGCTCTG–3′, Rev 5′–TTCTTGCGGAAGGTCAGG–3′, LPA2 Fwd 5′–TCAACACGGGACCTAATACC–3′, Rev 5′–GGCGAACATAGCCAAAGAT–3′, and LPA3 Fwd 5′–CAGGGAGGGCAGTATGTT–3′, Rev 5′–CCAGAATGGCTGTGAAGAT–3′. GAPDH primers used were Fwd 5′–ACCACAGTCCATGCCA TCAC–3′ and Rev 5′–TCCACCACCCTGTTGCTGTA–3′.

### 4.4. Cell Migration Assay

Cell migration was studied by the Boyden chamber method [58]. Briefly, test agents were placed in the lower chamber and an 8-μm polycarbonate filter was placed between the upper and lower chambers. Rat aortic smooth muscle cells (RASMCs) were trypsinized and resuspended at a concentration of 1x10^5^ cells/mL in DMEM containing 0.5% bovine serum albumin (BSA) and 100 μL (1x10^5^ cells) was added to the upper chamber. After incubation for 6 h at 37°C in a 5% CO2 incubator, filters were removed and the cells on the top surface of the membrane were removed with a cotton swab. The membranes were washed with phosphate-buffered saline, fixed with methanol and stained with hematoxylin and eosin. The number of cells that had migrated to the lower surface was determined by counting the cells in four randomly chosen places under a microscope at ×400 magnification. The migration assay was performed in at least three independent experiments.

### 4.5. Measurement of ERK and p38MAPK Activation

To monitor ERK and p38 MAPK activation, SDS-PAGE was performed, followed by Western blot analysis with specific antibodies recognizing phospho-ERK, total ERK, and phospho-p38 MAPK, and total p38 MAPK following the manufacturer’s instructions. Briefly, treatment of RASMCs with various agents was terminated by addition of trichloroacetic acid, and the total cellular proteins were extracted with SDS-sample buffer [31]. An equal amount of protein (5 μg) was resolved by SDS-PAGE and transferred to Nitrocellulose Membrane (Pierce). To determine ERK and p38 MAPK phosphorylation, the membrane was incubated with each phosphospecific antibody. Immunoreactivity was visualized using an enhanced ECL detection kit (Pierce) and exposed to radiographic film. The blots were scanned and quantified by Gelpro32 (Beta 4.02 version for Windows).

### 4.6. Statistical Analysis

Data were presented as mean ±S.E.M, statistical significance was determined by Student’s t-tests for comparisons between pairs of groups and by ANOVA for multiple comparisons. P<0.05 was considered to be statistically significant.
